# Integrating infodemiology principles into public health curricula: a qualitative exploration of emerging educational needs

**DOI:** 10.3389/fmed.2026.1812926

**Published:** 2026-05-22

**Authors:** Miranda Bosse, Kaitlin Tager, Elizabeth Gould, Maanasa Kanimilli, Emily Burke

**Affiliations:** 1 Association of Schools and Programs of Public Health, Washington, DC, United States; 2 The de Beaumont Foundation, Bethesda, MD, United States

**Keywords:** academic public health, disinformation, health communication, infodemiology, Master of Public Health (MPH) curriculum, misinformation

## Abstract

**Background:**

The rapid expansion of digital information ecosystems has intensified the spread of health-related misinformation and disinformation, contributing to public mistrust and adverse health outcomes. Infodemiology, defined as the study of how information spreads and influences health behaviors, has emerged as a critical domain for public health practice. Despite its growing relevance, infodemiology remains inconsistently defined and is not systematically integrated into Master of Public Health (MPH) curricula. This study explores how infodemiology principles are currently taught and identifies opportunities to strengthen their integration into public health education.

**Methods:**

A qualitative study was conducted using three virtual focus groups with faculty from CEPH-accredited schools and programs of public health (*n* = 16 participants representing 13 institutions across the United States). Participants were recruited through ASPPH networks based on involvement in health communication, health behavior or related teaching areas. Focus groups were conducted between August and October 2025, recorded, transcribed, and analyzed using line-by-line coding. A total of 24 codes were synthesized into six overarching themes through cross-group thematic analysis.

**Results:**

Although infodemiology is rarely explicitly labeled within MPH curricula, its core principles—such as media literacy, misinformation and disinformation analysis, audience engagement, and trust-building—are widely embedded across MPH coursework. Six themes emerged: (1) infodemiology as a cross-cutting and interdisciplinary domain; (2) widespread but implicit integration within existing teaching practices; (3) structural and organizational constraints, including limited curricular space and competing priorities; (4) increasing importance of technology, digital platforms, and analytic skills; (5) gaps in applied, workforce-ready training; and (6) the influence of sociopolitical contexts on instructional approaches. Participants emphasized the need for clearer conceptual framing, alignment with accreditation competencies, faculty development, and flexible, modular instructional resources to support integration.

**Discussion:**

Infodemiology represents a critical framework for modernizing public health education and strengthening workforce preparedness. Its integration into MPH curricula does not require entirely new structures but rather intentional alignment with existing competencies and expansion of applied learning opportunities. Embedding infodemiology principles within MPH curricula can better equip future public health professionals to navigate complex information environments and respond effectively to misinformation in practice.

## 1 Introduction

The rapid expansion of digital information ecosystems has intensified the spread of health-related misinformation and disinformation across the United States. Misinformation, defined as inaccurate or misleading information shared without intent to deceive, and disinformation, information deliberately disseminated to mislead or manipulate, have become pervasive features of contemporary information environments (1, 2). An estimated 30% of U.S. adults now report relying on social media sites, such as TikTok, Instagram, Facebook, and Twitter (X), as their primary source of news, reflecting a broader shift away from traditional media, including television, radio, and newspapers (3, 4). Because social media is largely driven by user-generated content, individuals can rapidly disseminate information regardless of accuracy, enabling false or misleading claims to circulate widely (4). Research further indicates that non-expert influencers often gain disproportionate reach by using strategies that grant their content a “competitive advantage” in attention and visibility, thereby significantly impacting the way information, regardless of accuracy, spreads (5, 6). Media analyses of digital content show that individuals and organizations with large social media audiences have heightened responsibility to ensure the accuracy of the health information they share (7). Simultaneously, fact-checking mechanisms struggle to keep pace with content volume, citizen-led “social fact-checking” has been shown to be ineffective (8), and algorithmic systems further amplify misinformation by rewarding novelty, emotional engagement, and repeated exposure over accuracy (9, 10).

These dynamics stress the urgent need for systematic approaches to understanding how health information spreads, influences thoughts and behavior, and shapes public trust, particularly during public health emergencies (11). The World Health Organization (WHO) defines an “infodemic” as an overabundance of accurate and inaccurate information that undermines informed decision-making by spreading confusion and amplifying viral falsehoods (12, 13). A response to infodemics, the field of infodemiology applies principles of epidemiology to examine the relationship between information environments, beliefs, and public health outcomes, with particular emphasis to misinformation risks and its effects on individuals, communities, and health systems (1, 2). Infodemiology aims to understand the distribution of information, especially in online environments, and how such information informs public health and policy (14). Understanding this field is imperative as infodemics contribute directly to patient mistrust, heighten public anxiety, strain clinical interactions, and increase demands on overburdened healthcare professionals, making infodemiology increasingly important for public health and population health (11, 15). These challenges are further compounded by varying levels of digital health literacy, a critical determinant of an individuals’ ability to access, evaluate, and apply digital health information and a key factor shaping vulnerability to misinformation in online ecosystems (16).

Recognizing the need to prepare public health professionals to practice infodemiology, the study of the spread of information, with the ultimate goal of improving public health, the de Beaumont Foundation and The Public Good Projects (PGP) developed the Infodemiology Training Program which offers applied, self-paced learning tailored to public health practitioners (1, 17). The Association of Schools and Programs of Public Health (ASPPH) then examined how these training objectives align with the Council on Education for Public Health (CEPH) Master of Public Health (MPH) Foundational Competencies to explore classroom and curricular application (18). This work, informed by focus groups with CEPH-accredited schools and programs of public health, generated practical insights for curricular integration, institutional variation, and workforce development. The resulting findings and recommendations highlight pathways for integrating infodemiology into academic public health.

## 2 Materials and methods

ASPPH conducted three focus groups to determine how principles of infodemiology are currently being taught within schools and programs of public health and to explore opportunities for further integration within MPH curricula. Participants were identified through a search of the ASPPH Academic Program Finder (APF) database, a comprehensive, searchable tool enabling individuals to explore accredited schools and programs of public health by area of study, location, and delivery method. A systematic review of the APF database identified 27 CEPH-accredited schools and programs of public health offering certificate or degree programs related to infodemiology, including health communication, misinformation, and crisis communication.

Participants were recruited via email outreach to representatives from ASPPH’s Academic Affairs Section, who oversee educational programming within each member institution (*n* = 27). Secondary recruitment emails were sent to faculty identified by Academic Affairs representatives who teach courses in health communication, crisis communication, digital health communication, or related subjects (*n* = 13). Recruitment for a third focus group expanded to include outreach to faculty at public universities teaching courses related to health communication, misinformation, communication theory, and behavior change (*n* = 19). Table 1 provides a summary of the academic institutions included in the three infodemiology focus groups, highlighting the geographic diversity and institutional representation across public health schools and programs through ASPPH member institutions. Three focus groups were conducted including six, three, and seven participants, representing 13 ASPPH-member schools and programs of public health, with two schools represented by two participants each.

**TABLE 1:** U.S. regions of participating institutions.

Name of Institution	Region
Boston University School of Public Health	Northeast
Cornell University Graduate Public Health Program	Northeast
Colorado School of Public Health	West
CUNY School of Public Health and Health Policy	Northeast
Emory University Rollins School of Public Health	South
Georgia Southern University Jiann-Ping Hsu College of Public Health	South
Oregon State University College of Public Health	West
San Diego State University School of Public Health	West
Tulane University Celia Scott Weatherhead School of Public Health and Tropical Medicine	South
University of Maryland School of Public Health	Northeast
University of South Florida College of Public Health	South
University of Utah School of Medicine, Division of Public Health	West
University of Washington School of Public Health	West

It provides a summary of academic institutions included in the three Infodemiology focus groups. Each entry lists the institution’s name and geographic region. This distribution highlights the geographic diversity and institutional representation across public health schools and programs through ASPPH member institutions.

A standardized focus group facilitation guide was used across all sessions to prompt discussion on real-world teaching practices, infodemiology in curriculum, and future curriculum directions. Focus groups were conducted virtually between August and October 2025, lasting approximately 90 min, and were recorded and transcribed with participant permission.

Each focus group transcript was analyzed, coding line by line to identify key themes. A total of 24 codes were consolidated into six themes through cross-group comparison, prioritizing patterns that were consistently raised across participants. Table 2 provides a comprehensive table that maps each analytic theme to its associated codes, along with definitions for all codes used in the qualitative analysis.

**TABLE 2:** Themes and contributing codes.

Theme	Codes
**Theme 1: Positioning Infodemiology Within Public Health Education and Practice**	● Applied Practice Orientation ● Core Competencies and Accreditation ● Health Communication Discipline Positioning ● Interdisciplinary Roles ● Public Health Field ● Terminology and Scope ● Trust and Credibility
**Theme 2: Existing Teaching Practices That Already Incorporate Infodemiology Principles**	● Audience and Culture ● Engaged Community Projects ● Essential Communication Skills ● Mis- and Disinformation Content ● Media Literacy and Critical Thinking ● Real World Examples and Cases ● Student Reflection and Application ● Writing and Message Basics
**Theme 3: Structural and Organizational Conditions Shaping Infodemiology Integration**	● Course Design Format ● Curricular Space and Integration ● Leadership and Advanced Training ● Mis- and Disinformation Challenges ● Sociopolitical Constraints and Conflict ● Trust and Credibility
**Theme 4: Technology and Digital Information Systems in Public Health Training**	● Applied Practice Orientation ● Audience and Culture ● Mis- and Disinformation Challenges ● Technology, AI, and Information Systems ● Trust and Credibility ● Tools and Analytic Capacity ● Social Marketing and Partnerships
**Theme 5: Workforce Preparedness and Applied Practice Gaps**	● Applied Practice Orientation ● Essential Communication Skills ● Interdisciplinary Roles ● Media Literacy and Critical Thinking ● Public Health Field ● Real World Examples and Cases ● Student Reflection and Application ● Writing and Message Basics ● Workforce Preparedness Gap
**Theme 6: Sociopolitical Context and Constraints on Teaching**	● Curricular Space and Integration ● Essential Communication Skills ● Media Literacy and Critical Thinking ● Mis- and Disinformation Content ● Mis- and Disinformation Challenges ● Sociopolitical Constraints and Conflict ● Terminology and Scope ● Trust and Credibility

Appendix is designed to offer transparency into the analytic process and to illustrate how specific codes supported the development of each thematic category. Codes were refined and unified across the three focus groups to ensure consistency, and definitions were developed to clarify the meaning and analytic boundaries of each code. Together, the table and definitions demonstrate how participant insights were systematically organized and interpreted to inform the findings presented in this report. Some codes appear in more than one thematic category because they captured ideas or experiences that were relevant across multiple dimensions of the analysis. Individual codes may contribute to several themes when participants discuss the same concept in different contexts or when the coded idea bridges conceptual, structural, or pedagogical issues. Codes were therefore mapped based on their analytic contribution rather than restricted to a single theme, ensuring that the thematic structure accurately represents the complexity and interconnectedness of participant perspectives. **Code Definitions:** ● **Applied Practice Orientation:** Need for applied, intervention-focused skills ● **Audience and Culture:** Attention to audience, culture, and context ● **Core Competencies and Accreditation**: Leveraging competencies and accreditation structures ● **Course Design Format:** Course structure, modalities, and scaffolding ● **Curricular Space and Integration:** Where infodemiology fits in existing curricula ● **Engaged Community Projects:** Project-based learning with external partners ● **Essential Communication Skills:** Foundational skills that students need ● **Health Communication Discipline Positioning:** Health communication as the primary disciplinary home ● **Interdisciplinary Roles:** Interdisciplinary collaboration and diverse communication roles ● **Leadership and Advanced Training:** Role of leadership and advanced degrees in communication ● **Media Literacy and Critical Thinking:** Media literacy and critical evaluation of information ● **Mis- and Disinformation Content**: Misinformation and disinformation as core course content ● **Mis- and Disinformation Challenges**: Difficulty defining and addressing mis- and disinformation challenges ● **Real World Examples and Cases**: Use of real-world and community-based examples in teaching ● **Public Health Field:** Field-wide needs ● **Social Marketing and Partnerships:** Use of social marketing and community partnerships ● **Sociopolitical Constraints and Conflict**: Operating under political constraints and managing conflict ● **Student Reflection and Application:** Reflection and application to students’ own practice ● **Technology, AI, and Information Systems**: Centrality of AI, digital platforms, and systems thinking ● **Terminology and Scope:** Concerns about terminology, jargon, and the scope of infodemiology ● **Trust and Credibility:** Trust in public health institutions and messengers ● **Tool Access and Media Monitoring:** Access to media monitoring and analytic tools ● **Workforce Preparedness Gap:** Gap between academic preparation and workforce needs ● **Writing and Message Basics**: Foundational writing and message design skills.

## 3 Results

Analysis of the three transcripts highlighted consistent themes in how infodemiology-related content is taught, how the specialty is conceptualized within academic public health, and what supports are needed for broader integration into MPH curricula. Although few participants noted that their institutions rarely label this content as “infodemiology,” they consistently reported that core concepts, such as media literacy, misinformation and disinformation, audience analysis, trust-building, and applied communication casework, are embedded within their coursework. These topics are being taught across health communications, behavioral sciences, crisis communications, and community engagement courses.

Participants agreed that understanding information systems and communication behaviors is essential for contemporary public health practice. Participants emphasized that meaningful integration of infodemiology into MPH curricula requires intentional support, including clear conceptual framing of the specialty, alignment with CEPH competencies, flexible and modular instructional materials, and opportunities for faculty development and training. Given workload demands, evolving technology and sociopolitical dynamics, the incorporation of infodemiology principles into MPH curricula must be done in a thoughtful and intentional manner.

### 3.1 Positioning infodemiology within education for public health and practice

Across the three focus groups, participants consistently framed infodemiology as relevant to public health practice, though the term itself remains unfamiliar and underdeveloped in MPH curricula. Participants recognized that its conceptual foundations, including how information is produced, disseminated, contested, and acted upon, are embedded across courses in communications, behavioral science, and in practice. However, these elements are not intentionally integrated, reflecting both the emerging nature of the field and the lack of shared vocabulary to define its scope, boundaries, and pedagogical purpose.

Participants emphasized that contemporary public health practice is inseparable from the information landscapes that shape risk, trust, and decision-making processes. Misinformation and disinformation were described as central to classroom discussion and unavoidable topics when preparing students for field work. Participants stressed that infodemiology is not merely an academic add-on but an analytic lens that helps practitioners interpret complex communication environments, respond to information threats, and navigate sociopolitical contexts. Classroom examples illustrate how academic public health faculty teach applied competencies such as critical evaluation, respectful engagement, and evidence-informed response.

Despite academic alignment, participants expressed uncertainty about how infodemiology should be positioned within MPH curricula. Rather than situating it solely as a subspecialty of health communications or informatics, participants emphasized its interdisciplinary nature and described it as a cross-cutting domain, similar to systems thinking, positioning it as a unifying competency that builds on existing strengths while addressing emerging practice needs. Participants also connected infodemiology to public health credibility and community trust, emphasizing that effective communication requires understanding community beliefs and how trust is built or broken.

Participants repeatedly connected infodemiology to existing accreditation expectations within CEPH’s MPH Foundational Competencies, viewing it as a means to strengthen required skills in communication, evidence-based practice, leadership, professionalism, cultural competence, and systems thinking. Rather than representing an additional curricular burden, it was framed to make explicit and more intentional competencies that are already implicitly embedded in accreditation expectations. Overall, participants positioned infodemiology as an essential, cross-cutting domain that requires clearer definitions and competency-level framing to support intentional instruction and evaluation.

### 3.2 Existing teaching practices incorporate infodemiology principles

Participants across all three focus groups emphasized that their teaching incorporates many of the core principles of infodemiology, even when the term is not explicitly used. Courses routinely address how people encounter, interpret, and act on health information, particularly in the context of misinformation, outbreaks, and public health controversies. Participants described extensive use of real-world materials, including websites, social media content, and campaign artifacts to expose students to authentic information landscapes. Students bring real-time examples from social media and news outlets into the classroom, which faculty integrate into case discussions, critiques, and analysis exercises aligned with infodemiology’s focus on information patterns and their effects on population-level understanding and behavior. Many instructors situate their teaching within formats that mirror the tasks students will encounter in public health practice, such as applied, project-based learning tied to local public health issues. Such approaches require students to understand audiences, develop strategies, and navigate complex information ecosystems, reinforcing infodemiology principles.

Participants also highlighted communication skill-building as foundational to public health practice, emphasizing the need for skill-building in writing, message design, audience analysis, and cultural sensitivity. They noted the importance of helping students identify trusted information sources and tailor messages to specific audiences. Reflection and application exercises further connect course content to broader information dynamics and help students translate expert knowledge into accessible, trustworthy information.

Taken together, these accounts show that many MPH programs possess instructional infrastructure that supports infodemiology-related learning: real-world examples, applied projects, analytic exercises, and skill-building in message design and audience analysis. The opportunity lies in naming, connecting, and scaffolding these existing elements through shared definitions, learning objectives, and intentional curricular design.

### 3.3 Structural and organizational conditions shaping infodemiology integration

While participants were enthusiastic about the value of infodemiology, they were equally candid about the structural constraints shaping curricular change. Participants described MPH curricula as dense, with limited flexibility to add new content without displacing existing material. Faculty must balance competing priorities, and the underlying concern was clear: any proposal to expand infodemiology must contend with existing course structures, accreditation requirements, credit hour limitations, and must evolve to address technological and sociopolitical contexts.

Participants noted that controversial topics like misinformation, vaccines, and public trust can be difficult to address openly, particularly in politically polarized settings. Participants also discussed the emotional and pedagogical labor involved in facilitating such nuanced discussion, with these challenges being heightened in online learning environments.

At the same time, participants framed infodemiology integration as central to workforce preparation, creating both pressure and justification for change. Participants emphasized the urgent need for education for public health to move beyond conceptual discussions of infodemiology toward applied training that prepares students to respond to rapidly evolving information environments in real-world settings. This perspective positions infodemiology not as a theoretical add-on, but as applied training that bridges the gap between understanding information ecosystems and acting within them. Alignment with workforce needs and accreditation competencies was seen as a critical lever for overcoming structural barriers, in addition to seeing infodemiology as a potential asset for recruitment and program distinctiveness, suggesting that programs could leverage infodemiology as a marker of innovation and responsiveness to current public health challenges.

This theme illustrates that structural realities may slow or constrain change; however, alignment with the MPH Foundational Competencies and workforce needs can provide a powerful argument for integrating infodemiology into MPH curricula.

### 3.4 Technology, information systems, and analytic skills for public health communication

Participants consistently linked infodemiology to the technological systems through which information flows, including social media platforms and AI-generated content. Participants recognized that preparing students for this reality demands foundational literacy in how technology shapes the conditions of communication like exposure, interpretation, and trust.

Participants expressed varying levels of comfort and capacity in teaching about rapidly evolving technologies. They described prior exposure to media analysis approaches but noted uncertainty about available resources and effective use, underscoring the need for brief, targeted training to build confidence and support ongoing awareness of how public narratives evolve. Participants consistently emphasized the importance of distinguishing between conceptual literacy, understanding how technologies function and influence infodemics, and specialized technical training, which may be difficult to implement broadly given existing curricular and institutional constraints.

Participants expressed that technology is integral to infodemiology, both as a defining feature of the current information landscape and a source of student interest and anxiety. However, they stressed that teaching in this space must be realistic about institutional capacity and practice settings, and therefore effective teaching must provide adaptive analytic approaches that prepare students to critically assess information ecosystems, regardless of the tools available in a given job or public health practice setting.

### 3.5 Workforce preparedness and applied practice gaps

Across all focus groups, participants emphasized that infodemiology is fundamentally an applied skillset essential to public health practice. They expressed concern that students often lack opportunities to practice intervening in real information ecosystems, despite exposure to relevant concepts. They emphasized that MPH curricula does not always equip students with the applied skills needed to operate within professional landscapes where misinformation and disinformation spread rapidly, communication expectations are high, and practitioners must make quick, strategic decisions under uncertainty. Participants noted a persistent gap between classroom instruction and the demands of public health practice, as MPH graduates are increasingly expected to contribute immediately to social media monitoring and community messaging, yet many learn these skills on the job.

Participants also discussed variability in students’ baseline communication skills, underscoring the need for curricular scaffolding that addresses concepts such as message framing, persuasive writing, and adapting content for diverse audiences. Participants stressed that robust infodemiology training must go beyond teaching students *what* misinformation and disinformation are to teaching them *how* to intervene: conducting situational assessments, identifying trusted messengers, designing communication strategies, and navigating contested sociopolitical environments. These insights position infodemiology as a critical element of workforce development, implying that experiential learning, simulations, and partnerships with practice organizations will be central to closing the applied practice gap.

### 3.6 Sociopolitical context and constraints on teaching infodemiology

Participants emphasized the sociopolitical environment in which infodemiology is taught. Increasing politicization of public health shapes instructional choices, particularly in environments where discussions of misinformation or trust are perceived as partisan. This constraint shapes not only course content but instructional strategy, requiring instructors to balance academic rigor with personal and institutional safety considerations. Participants described adapting to such situations by anchoring discussions in evidence and theory while carefully managing classroom dynamics and varying perspectives, a strategy that allows them to maintain academic integrity while reducing exposure to conflict in the classroom.

These constraints have important implications for curriculum design, demonstrating the need for institutional support, including clarity around academic freedom, pedagogical training, and conflict management. An infodemiology curriculum developed for nationwide adoption must include content on the politics of information while remaining adaptable to sociopolitical pressures that vary across institutional and geographic contexts. Understanding and learning how to navigate the constraints imposed by polarized environments is central to preparing students for the realities of contemporary public health practice.

## 4 Cross-theme synthesis

The six themes illustrate how faculty conceptualize infodemiology, the pedagogical and structural conditions that shape its integration, and the implications for preparing a public health workforce able to operate within complex information ecosystems. Across focus groups, participants described teaching practices and workforce expectations that closely align with infodemiology principles, while also identifying structural, political, and conceptual barriers that limit the specialty’s formal recognition and intentional integration.

Across themes, infodemiology emerges less as a discrete specialty than as a natural extension of existing competencies, evidence-based decision-making, systems thinking, and cultural competence, positioning it as a framework that unifies concepts already being taught. This perspective suggests that the question facing schools and programs of public health is not *whether* infodemiology belongs in the curriculum, but *how* to conceptualize and structure it so that students develop both foundational knowledge and applied skills.

The connections across Themes 1 and 2 highlight the fact that instructors are already teaching infodemiology principles; however, participants identified a gap in applied learning, particularly in navigating contested narratives, responding to misinformation, and working within real-world constraints. Infodemiology thus becomes a bridge between conceptual knowledge and practice readiness by providing a framework for moving beyond awareness toward action.

Themes 3 and 4 reveal structural and organizational conditions that further shape potential integration. Participants noted that dense curricula, accreditation requirements, workload, institutional politics, and rapidly evolving technology constrain the addition of new content, even with clear perceived need. Such constraints underscore the need for flexible, modular resources and faculty development supports that lower the burden of integrating new content. By utilizing the CEPH MPH Foundational Competencies as strategic leverage for integration, participants argued that infodemiology strengthens, rather than competes with, existing expectations around communication, evidence-based practice, leadership, and systems thinking.

Themes 5 and 6 deepen this picture by emphasizing the technological demands of the public health workforce and the sociopolitical conditions under which teaching occurs. Graduates must be prepared to intervene in real-time information crises, interpret misinformation and disinformation, design response strategies, and engage communities across varied communication landscapes, but there remains a consistent gap between conceptual coursework and the applied competencies practitioners need. Simultaneously, the politicization of public health requires careful pedagogical navigation and institutional support, signifying that infodemiology education cannot be standardized in a one-size-fits-all manner but must remain adaptable to institutional, technological, and political contexts.

The themes present a coherent narrative that schools and programs of public health are poised to integrate infodemiology principles with intention but need conceptual clarity, institutional support, faculty training, and flexible instructional materials to do so effectively. Infodemiology’s value lies in its ability to connect elements of academic public health and practice, including communication, trust, behavior, technology, and politics, into a unified framework that reflects the conditions graduates will encounter in the field. Embedding infodemiology into curricula provides an opportunity to modernize academic public health, closely aligning with accreditation standards, workforce needs, and the evolving realities of communication in a polarized, digitally mediated world.

## 5 Recommendations: integrating infodemiology into MPH curricula

Focus group findings point to clear opportunities for designing an infodemiology curriculum that is responsive to institutional constraints while aligned with the realities of contemporary public health practice. The primary challenge facing infodemiology education is not the absence of relevant teaching but rather the lack of conceptual clarity and applied scaffolding across MPH competencies and curricula. Recommendations therefore emphasize making practice explicit, aligning curricular innovation with institutional realities and strengthening links between academic and workforce expectations in public health.

Curriculum development should begin with a systematic assessment of existing coursework and pedagogical approaches by faculty across departments and public health practitioners, to identify where infodemiology-related concepts are already being taught. Anchoring infodemiology within the CEPH MPH Foundational Competencies, including communication, evidence-based decision-making, systems thinking, leadership, and cultural competence, was also seen as essential for facilitating integration into already-full curricula and aligning with accreditation requirements. Establishing a shared definition and conceptual framework that maps information flows, behavioral and social responses, trust, technology, and public health interventions would further promote consistent understanding across institutions. Sustainable implementation also requires leadership buy-in, achieved through clear framing of infodemiology’s value to MPH graduates, flexible integration options, and institutional supports such as faculty development and tools. Participants underscored the need for ongoing faculty training, including workshops, discussion guides, and strategies for managing contentious classroom dynamics, as well as opportunities for continued cross-institutional dialogue to build confidence, maintain instructional quality, and adapt to rapidly evolving information environments.

Participants recommended developing instructional materials that can be easily incorporated into existing courses with minimal restructuring. Recommended materials include concise conceptual modules, real-world case studies, and practice-based assignments such as rapid response exercises, message revision tasks, narrative analysis, and audience trust analysis. They also stressed the sociopolitical nature of infodemiology, emphasizing the need for curriculum materials to directly address sociopolitical context by offering guidance for teaching in restrictive political environments, using neutral framing grounded in theory and evidence, and emphasizing ethical responsibilities when navigating contested information spaces. Such preparation enables both faculty and students to engage thoughtfully and safely across varied institutional and political contexts.

Finally, focus group participants underlined the importance of embedding workforce-ready, applied learning experiences throughout the curriculum, informed by ongoing consultation with the field to ensure relevance to real-world public health roles. Recommended experiences include simulations of emerging misinformation events, partnerships with local health departments or community organizations, and assignments that require drafting or evaluating public health messages. A distributed, cross-course integration approach that integrates infodemiology with health communications, behavioral and social sciences, leadership, policy, and community engagement, can reinforce learning over time and reduce the burden on any single course. Conclusion

Academic public health sits at a pivotal moment in which individuals across schools and programs of public health recognize that information ecosystems and public trust are central determinants of population health outcomes. Findings from this qualitative analysis demonstrate that while infodemiology is not widely taught as a standalone specialty, its core principles are embedded throughout MPH curricula. The six focus group themes reveal both the readiness and the challenges regarding infodemiology curricular integration including the need for conceptual clarity, adaptable instructional materials, variable institutional capacity, and evolving technological and sociopolitical pressures, indicating the importance of flexible, evidence-based resources that can be applied across diverse academic settings.

Together, the findings suggest that integrating infodemiology into MPH curricula is an opportunity to modernize education for public health. The recommendations offer a roadmap for designing a flexible, practice-oriented, and equitable approach to infodemiology education, positioning schools and programs of public health to prepare graduates with the skills necessary to navigate and shape the information ecosystems that profoundly influence public health practice and population health outcomes.

## Data Availability

The original contributions presented in this study are included in this article/supplementary material, further inquiries can be directed to the corresponding author.
